# RPRD1A stabilizes NRF2 and aggravates HCC progression through competing with p62 for TRIM21 binding

**DOI:** 10.1038/s41419-021-04447-4

**Published:** 2021-12-17

**Authors:** Xiaofan Feng, Tianyi Jiang, Chun Yang, Shujie Pang, Zhiwen Ding, Heping Hu, Hui Wang, Liwei Dong, Ning Yang

**Affiliations:** 1 National Center for Liver Cancer, the Second Military Medical University, 201805 Shanghai, P.R. China; 2 Department of Surgery, Eastern Hepatobiliary Surgery Hospital, Second Military Medical University, 200438 Shanghai, P.R. China; 3 Children’s Hospital of Soochow University, 215025 Suzhou, P. R. China; 4 Department of Hepatic Surgery, Fudan University Shanghai Cancer Center, 200032 Shanghai, P.R. China; 5 Department of Hepatobiliary Medicine, Eastern Hepatobiliary Surgery Hospital, Second Military Medical University, 200438 Shanghai, P. R. China

**Keywords:** Liver cancer, Oncogenes

## Abstract

NRF2 is the master transcriptional activator of cytoprotective genes and Kelch-like ECH-associated protein 1 (Keap1), a biosensor for electrophiles and oxidation, promotes NRF2 degradation in unstressed conditions. SQSTM1/p62, an oncogenic protein aberrantly accumulated in hepatocellular carcinoma (HCC), binds and sequestrates Keap1, leading to the prevention of NRF2 degradation. Here, we show that p15INK4b-related sequence/regulation of nuclear pre-mRNA domain-containing protein 1A (RPRD1A) is highly expressed in HCC tumors and correlated with aggressive clinicopathological features. RPRD1A competitively interacts with TRIM21, an E3 ubiquitin ligase of p62, resulting in the decrease of p62 ubiquitination and the increased sequestration for Keap1. Therefore, RPRD1A enhances the nuclear translocation of NRF2, which induces gene expression for counteracting oxidative stress, maintaining cancer cells survival, and promoting HCC development. Moreover, disturbing the redox homeostasis of cancer cells by genetic knockdown of RPRD1A sensitizes cancer cells to platinum-induced cell death. Our study reveals RPRD1A is involved in the oxidative stress defense program and highlights the therapeutic benefits of targeting pathways that support antioxidation.

## Introduction

Worldwide, liver cancers are the third most common cause of cancer-related death [1], and hepatocellular carcinoma (HCC) accounts for the majority (~90%) of primary liver cancers [2]. Although surgical excision or transcatheter arterial chemoembolization (TACE) has been successfully applied in treating HCC, the prognosis of this disease remains dismal [3]. Many factors lead to the poor prognosis of HCC, including high frequency of recurrence or distant metastasis and high resistance to chemotherapy [4]. Therefore, deepening the understanding of the tumor progression mechanism of HCC will be beneficial for the development of effective intervention strategies.

Oxidative stress, which is represented by the raising of reactive oxygen species (ROS), disturbs the cellular redox-status and occurs throughout cancer development [5]. ROS plays a complex role on cancer biology. On one hand, the increased ROS levels are involved in the promotion and maintenance of tumorigenic cell signalings, which contribute to tumorigenesis, cancer progression and spreading by facilitating cell proliferation, survival, autophagy, and metastasis [6]. On the other hand, excessive ROS also results in anti-tumorigenic effects, inducing cell cycle arrest, and senescence [7]. In general, enhanced neutralization for ROS can protect normal cells from tumor transformation, or alternatively, an increased redox capacity may promote the proliferation of cancerous cells [8].

Tumor cells fight against oxidative damage through many ways, among all NRF2 is the master transcriptional activator in response to electrophiles and ROS [9]. NRF2 is mainly regulated at the protein level, and Kelch-like ECH-associated protein 1 (Keap1) is responsible for the degradation of NRF2 in unstressed conditions. Notably, redox-disrupting stimulus directly modifies Keap1 thiols and inactivates the Keap1 function, leading to the stabilization of NRF2, and induction of cytoprotective genes [10]. In addition, a series of intrinsic mediators have recently been discovered to interact with either Keap1 or NRF2, which impact the formation of the Keap1-NRF2 complex and the cellular antioxidative status [11]. One of the landmark discoveries regarding this is that SQSTM1/p62-dependent sequestration of Keap1, which leads to prolonged activation of NRF2 in a noncanonical, cysteine-independent manner [12]. It has been reported that the ubiquitin E3 ligase TRIM21 directly interacts with p62 and ubiquitylates it at the residue of K7, which inhibits the dimerization of p62 and its sequestration function for Keap1 [13]. However, how the p62- Keap1 axis is regulated, thereby activating NRF2 transcriptional activity in HCC remains elusive.

One acknowledged characteristic of HCC is the propensity to invade the vasculature within the liver. Portal vein tumor thrombosis (PVTT) is the most common form of macro-vascular invasion of HCC [14]. Patients with PVTT usually have aggressive disease symptoms, with an impaired liver function, higher recurrence rates, and very dismal prognosis [15]. However, the mechanism of PVTT formation remains largely unknown. To gain insight into the molecular events in HCC metastases, high-throughput sequencing of RNA was performed between paired HCC primary tissues and PVTT tissues. As a result, we identified that p15INK4b-related sequence/regulation of nuclear pre-mRNA domain-containing protein 1A (RPRD1A) was highly expressed in HCC tumors, especially in PVTT metastases. Mechanistically, RPRD1A interacted with TRIM21, enhancing p62 and Keap1 aggregation to promote the nuclear translocation of NRF2, which promoting HCC development.

## Results

### RPRD1A is frequently up-regulated in HCC and correlated with aggressive clinicopathological features

**Fig. 1:**
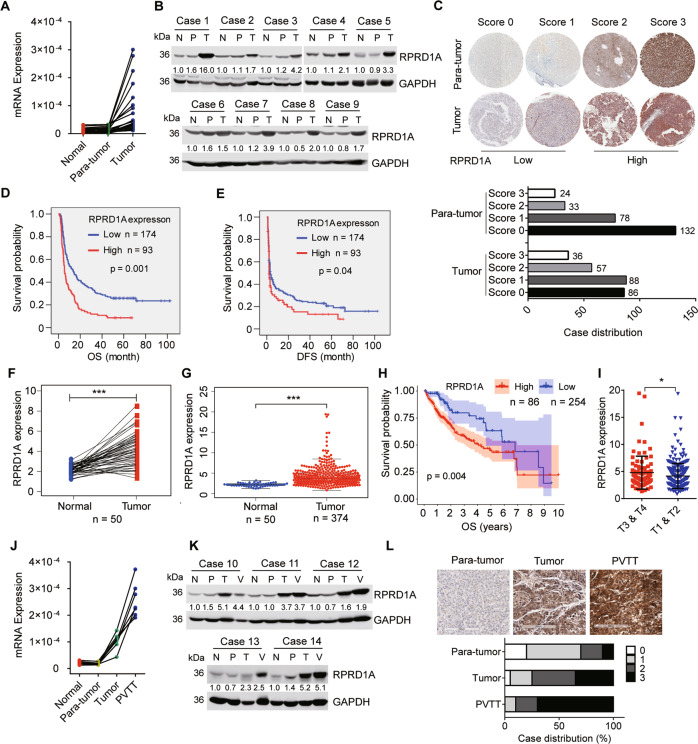
RPRD1A was overexpressed in HCC samples, and its overexpression correlated with aggressive clinicopathological features. **A** Real-time PCR (RT-PCR) analysis of RPRD1A mRNA expression in 24 paired primary tumor (T), para-tumor (P), and normal tissues (N) obtained from the Eastern Hepatobiliary Surgery Hospital (EHBH). **B** Western blotting analysis of RPRD1A protein expression in 9 paired HCC tumor, para-tumor, and normal tissues obtained from the EHBH. **C** Representative images of RPRD1A staining (score 0, 1, 2, 3) in HCC tissue microarray and the case distribution of different staining scores were shown. **D**, **E** (**D**) Overall survival (OS) and (**E**) disease-free survival (DFS) in an HCC cohort obtained from EHBH according to the RPRD1A expression, the log-rank test was used to determine significance. **F** RPRD1A expression in 50 paired HCC tumor and normal tissues from the TCGA database. **G** RPRD1A expression in 374 cases of HCC and 50 cases of normal tissues from the TCGA database. **H** Overall survival time in an HCC cohort with 343 cases obtained from the TCGA database according to the RPRD1A mRNA expression. **I** Expression of RPRD1A in tumor T1&T2 and T3&T4 in the database. **J**–**K** (**J**) RT-PCR and (**K**) western blotting analysis of RPRD1A in normal liver (N), para-tumor (P), primary tumor (T), and portal vein tumor thrombosis tissues (PVTT). **L** IHC staining and statistical analysis of the RPRD1A expression (score 0, 1, 2, 3) in PVTT, HCC, and para-tumor tissues. Scale bars, 200 μm. In panels **D**, **E**, **H**, *p* values were determined by the log-rank test. In panels **F**, **G**, **I**, the *p* values were determined by a two-tailed t-test. *p < 0.05, **p < 0.01, ***p < 0.001.

To profile the gene expression in HCC metastases, we carried out RNA-seq between paired HCC primary tissues and PVTT tissues and discovered RPRD1A up-regulated in PVTT tissues (supplementary file 1). To verify this bioinformatics finding, we examined the expression of RPRD1A in HCC tissues. Intriguingly, most cases of HCC showed much higher mRNA levels of RPRD1A than the corresponding para-tumor or normal liver tissues (Fig. 1A). Western blotting assays also revealed that RPRD1A up-regulated in HCC primary tissues (Fig. 1B). We further confirmed our observation in an expanded cohort of 267 HCC cases by immunohistochemistry (Fig. 1C, top). The results showed that 78.6% (210/267) para-tumor tissues presented low-expression (score 0 and 1) and 21.4% (57/267) presented high-expression (score 2 and 3) of RPRD1A, while the tumor tissues showed 65.2% (174/267) low-expression and 34.8% (93/267) high-expression (Fig. 1C, bottom).

Further, we analyzed the relationship between RPRD1A protein expression and clinicopathological features of HCC in high- and low-expression groups based on immunohistochemistry staining. As a result, RPRD1A positively correlated with tumor size, vein invasion, TNM stage and BCLC stage features (Table 1). Moreover, patients in RPRD1A high-expression group had a shorter overall survival (OS) and disease-free survival (DFS) (Fig. 1D, E). Consistently, analyses of the TCGA database revealed that HCC tumor tissues had higher mRNA expression of RPRD1A in both paired (Fig. 1F) and unpaired specimens (Fig. 1G). Based on the mRNA expression of RPRD1A, we divided HCC patients into RPRD1A high-expression (the top quarter, *n* = 86) and low-expression (*n* = 254) groups. Likewise, patients in RPRD1A high-expression group had a much shorter overall survival (Fig. 1H). Moreover, HCC with high TNM grade (T3 & T4) had much higher mRNA expression of RPRD1A (Fig. 1I), indicating RPRD1A positively correlated with HCC aggressive features. Indeed, real-time PCR and western blotting verified that PVTT tissues had a higher expression of RPRD1A than primary tumors and normal liver tissues (Fig. 1J, K). Furthermore, 20 pairs of HCC samples were subjected to immunohistochemical staining and the results showed that about 70% of PVTT presented a strong expression of RPRD1A (Fig. 1L). Altogether, these data demonstrated that RPRD1A was highly expressed in HCC tumors, especially in PVTT metastases, and its overexpression correlated with aggressive pathological features.

**Table 1:** Relationship between RPRD1A protein expression and clinicopathologic characteristics (*n* = 267).

Characteristics	No. patients	RPRD1A immunoreactivity		*P* value
	(%)	Low	High	
*Age, year*				0.073
≤49	149 (55.8)	91	58	
>49	118 (44.2)	83	35	
*Gender*				0.12
Male	234 (87.6)	149	85	
Female	33 (12.4)	25	8	
*HBs Ag*				0.372
Negative	20 (7.5)	14	6	
Positive	247 (92.5)	161	86	
*Tumor size (cm)*				0.011
≤5	46 (17.2)	37	9	
>5	221 (82.8)	137	84	
*Tumor number*				0.342
Single	245 (91.8)	161	84	
Multiple	22 (8.2)	13	9	
*AFP (ng/mL)*				0.5
≤400	79 (29.6)	51	28	
>400	188 (70.4)	123	65	
*Invasion*				0.121
Negative	183 (68.5)	124	59	
Positive	84 (31.5)	50	34	
*BCLC stage*				0. 010
A	39 (14.6)	33	6	
B	71 (26.6)	48	23	
C	157 (58.8)	93	64	
*Portal vein tumor emboli (PVTT)*				0.008
Negative	111 (41.6)	82	29	
Positive	156 (58.4)	92	64	
*Microscopic portal vein tumor thrombus (MI-PVTT)*				0.025
Negative	29 (10.9)	24	5	
Positive	238 (89.1)	150	88	
*TNM stage*				0.001
I & II	90 (33.7)	72	18	
III & IV	177 (66.3)	102	75	
*NRF2 staining*				0.019
Low	86 (32.2)	64	22	
High	181 (67.8)	110	71	

### RPRD1A promoted HCC progression both in vitro and in vivo

**Fig. 2:**
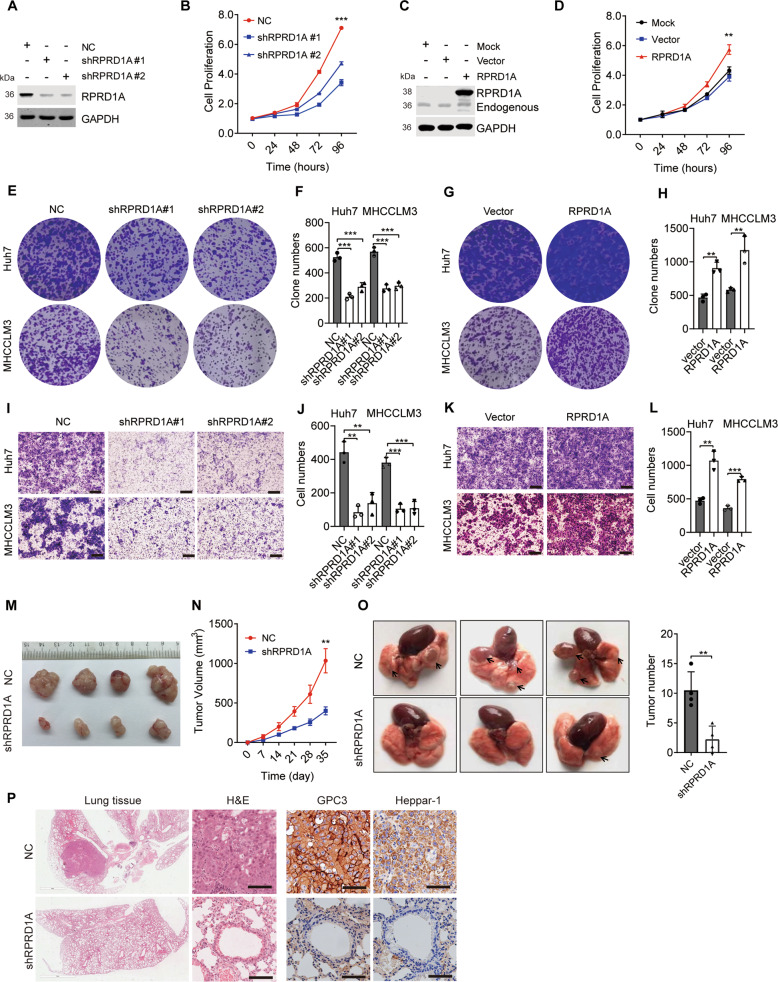
RPRD1A promoted HCC progression both in vitro and in vivo. **A** Western blotting detected the expression of RPRD1A in MHCCLM3 control (NC) and RPRD1A knockdown (shRPRD1A #1 and #2) cells. **B** Cell proliferation of MHCCLM3 NC and RPRD1A knockdown cells by CCK8 assay. **C** Western blotting detected the overexpression of RPRD1A by plasmids transfection in MHCCLM3 cells. **D** Cell proliferation of RPRD1A overexpressed MHCCLM3 cells by CCK8 assay. **E**–**H**, (**E**, **G**) Representative images and quantification (**F**, **H**) of plate colony formation assays in MHCCLM3-NC, MHCCLM3-shRPRD1A, Huh7-NC, Huh7-shRPRD1A, MHCCLM3-vector, MHCCLM3-RPRD1A, Huh7-vector, and Huh7-RPRD1A cells. **I-L**, (**I**, **K**) Representative images and quantification (**J**, **L**) of transwell migration assay in MHCCLM3-NC, MHCCLM3-shRPRD1A, Huh7-NC, Huh7-shRPRD1A, MHCCLM3-vector, MHCCLM3-RPRD1A, Huh7-vector and Huh7-RPRD1A cells. **M** Tumor gross images of MHCCLM3 NC- and shRPRD1A-derived subcutaneous tumors. **N** Growth curves of MHCCLM3 NC- and shRPRD1A-derived subcutaneous tumors. **O** Representative images and quantification of lung metastases derived from MHCCLM3 NC and shRPRD1A cells by tail vein injection. Arrows indicated the tumors. **P** Representative images of tumor pulmonary metastases with hematoxylin & eosin (H&E) and immunohistochemical (IHC) staining. IHC was stained to detect GPC3 and Heppar-1. The *p* values were determined by a two-tailed t-test. *p < 0.05, **p < 0.01, ***p < 0.001. Results are representative of at least three independent experiments.

Given that RPRD1A overexpressed in HCC and correlated with tumor invasion clinical characteristics, we postulated that RPRD1A would substantially affect the progression of HCC. To verify our postulation, we established RPRD1A stable knockdown and overexpression cells in two different HCC cell lines, MHCCLM3 and Huh7 cells (Fig. S1A). As expected, two independent shRNA sequences targeted RPRD1A (shRPRD1A) markedly suppressed HCC cell proliferation in vitro (Fig. 2A, B and Fig. S1B, S1C), while overexpression of RPRD1A increased cell proliferation (Fig. 2C, D and Fig. S1D, S1E). In addition, RPRD1A knockdown led to suppression of colony formation in both MHCCLM3 and Huh7 cells (Fig. 2E, F), while overexpression of RPRD1A intensified colony formation (Fig. 2G, H). To investigate the effects of RPRD1A on the tumor invasion capacity of HCC cells, transwell assays were performed. The results showed that knockdown of RPRD1A inhibited cell invasion (Fig. 2I, J) and overexpression of RPRD1A promoted it (Fig. 2K, L). Moreover, MHCCLM3 cells stably expressing shRPRD1A were inoculated into the flanks of nude mice, and the effect of RPRD1A on xenograft tumor growth was evaluated. RPRD1A-depletion significantly inhibited tumor growth in vivo compared with control cells (Fig. 2M, N). To assess the regulatory role of RPRD1A on tumor invasion and metastasis, MHCCLM3 cells stably expressing shRPRD1A or control cells were injected into the tail veins of nude mice. A significantly decreased number and size of pulmonary metastatic lesions were observed in knockdown cells (Fig. 2O). Further, we performed H&E and IHC staining of HCC markers to confirm that the metastases to the lung possessed HCC cell characteristics (Fig. 2P). Taken together, these data demonstrated that RPRD1A promoted HCC progression both in vitro and in vivo.

### RPRD1A decreases the intracellular ROS level and enhances the capacity to cope with oxidative stress

**Fig. 3:**
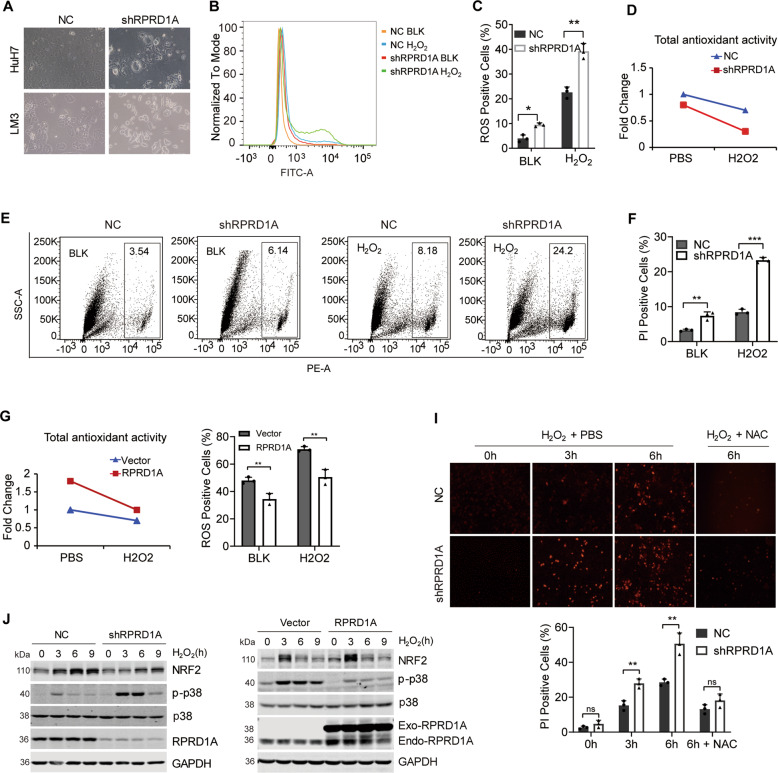
RPRD1A decreases the intracellular ROS level and enhances the capacity to cope with oxidative stress. **A** Optical microscope pictures of Huh7 and MHCCLM3 cells transfected with shRPRD1A and control lentivirus for 72 h. **B** Analysis of ROS levels by flow cytometry in MHCCLM3-NC and -shRPRD1A cells treated with 0.5 mM H_2_O_2_ for 4 h. **C**, Quantification of the ROS levels in (**B**). **D** Total antioxidant capacity was measured with a T-AOC assay kit in MHCCLM3-NC and -shRPRD1A cells treated with 0.5 mM H_2_O_2_ or PBS for 4 h. **E** ROS-mediated cell death was determined by flow cytometry. MHCCLM3-NC and -shRPRD1A cells were treated with 0.5 mM H_2_O_2_ for 9 h, and stained with PI for 20 min. The number of PI-positive cells was then observed and quantified. **F** Quantification of cell death in (**E**). **G** Total antioxidant capacity was measured with a T-AOC assay kit in RPRD1A overexpressed and control MHCCLM3 cells treated with 0.5 mM H_2_O_2_ or PBS for 4 h. **H** Quantification of the ROS levels in RPRD1A overexpressed and control MHCCLM3 cells treated with 0.5 mM H_2_O_2_ for 4 h by flow cytometry. **I** PI staining analysis and quantification in MHCCLM3-NC and -shRPRD1A cells treated with 0.5 mM H_2_O_2_ or 0.5 mM H_2_O_2_ plus 100 nM NAC for the indicated time. **J** Western blotting detected the expression of RPRD1A, NRF2, p-p38, and p38 in RPRD1A knockdown and overexpressed MHCCLM3 cells treated with 0.5 mM H_2_O_2_ for the indicated time. The *p* values were determined by a two-tailed t-test. *p < 0.05, **p < 0.01, ***p < 0.001, ns, not significant. Results are representative of at least three independent experiments.

When constructing the stable interfered RPRD1A HCC cell lines, we noticed that shRNA-expressing cells caused more cell death than the control cells at the first week (Fig. 3A). We also found that transient depletion of RPRD1A significantly increased ROS production in HCC cells, and accumulated much more ROS when treated with H_2_O_2_ (Fig. 3B, C). Thus, we speculated that RPRD1A might play an important role in antioxidative stress. To corroborate this theory, we evaluated the antioxidant ability by detecting GSH, the cellular major antioxidant, in shRPRD1A and NC expressing cells treated with or without H_2_O_2_. RPRD1A deletion in HCC cells induced a strong decrease in GSH/GSSG ratio, indicating an impaired antioxidant capacity (Fig. 3D). Consequently, RPRD1A knockdown caused more cell death of HCC cells at the baseline or treated with H_2_O_2_ (Fig. 3E, F). On the contrary, RPRD1A overexpression increased the cellular GSH/GSSG ratio (Fig. 3G) and protected cells from H_2_O_2_ treatment (Fig. 3H). Moreover, cell death caused by RPRD1A knockdown under H_2_O_2_ treatment could be rescued by the antioxidant N-acetyl-L-cysteine (NAC) (Fig. 3I), indicating that RPRD1A participated in the regulation of ROS-induced cell death. Further western blotting revealed that RPRD1A knockdown increased the phosphorylation of p38 MAPK (a biosensor indicating oxidative stress) and decreased the accumulation of NRF2 protein under H_2_O_2_ treatment, whereas overexpression of RPRD1A led the opposite phenomena (Fig. 3J). As NRF2 was the key regulator of cytoprotective gene and antioxidative reactions, we hypothesized that RPRD1A modulated the sensitivity to oxidative stress in HCC cells by affecting NRF2.

### RPRD1A inhibits ubiquitin-proteasome mediated degradation of NRF2

**Fig. 4:**
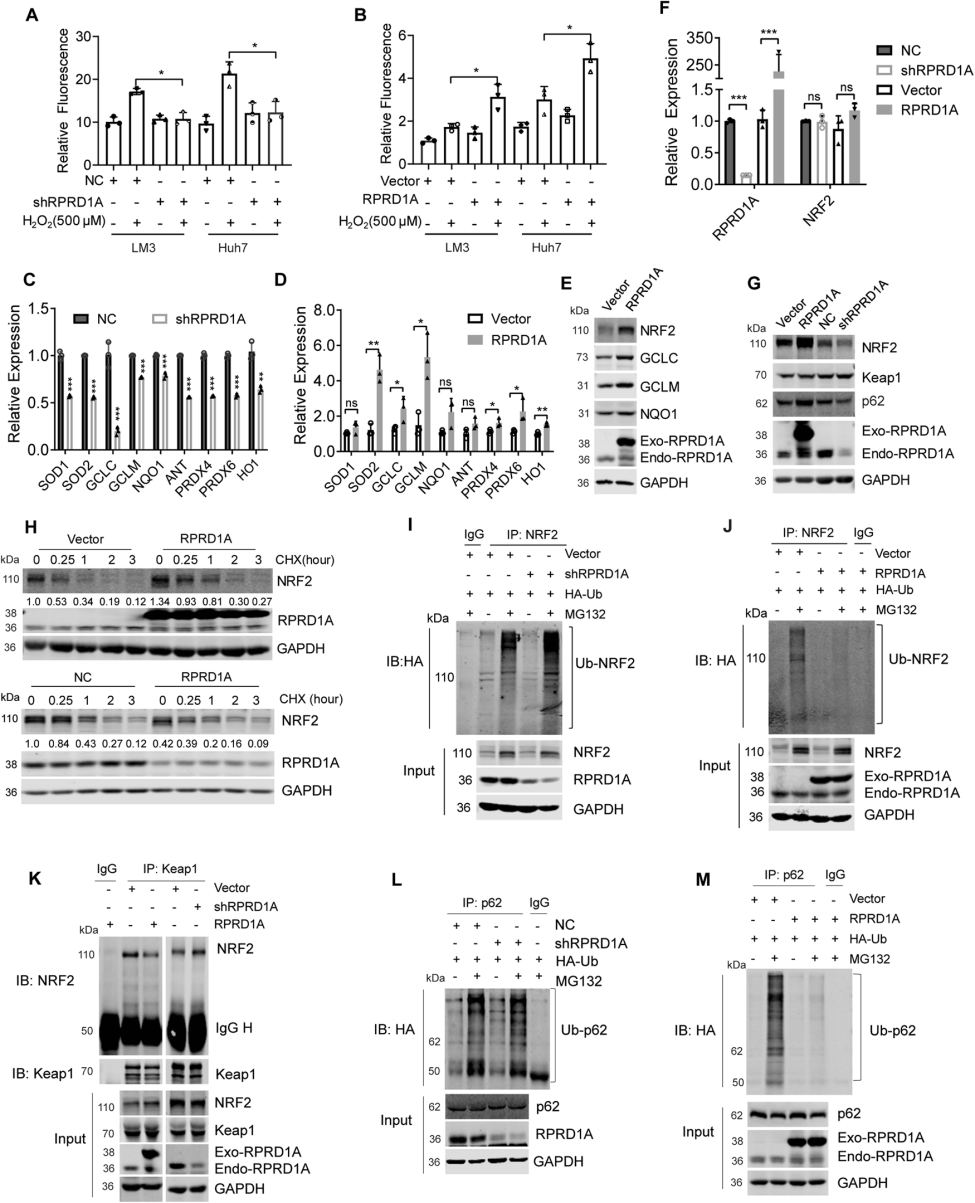
RPRD1A inhibits ubiquitin-proteasome mediated degradation of NRF2. **A**, **B** ARE-luciferase reporter activity assay in MHCCLM3-NC, MHCCLM3-shRPRD1A, MHCCLM3-vector and MHCCLM3-RPRD1A (**A**), Huh7-NC, Huh7-shRPRD1A, Huh7-vector and Huh7-RPRD1A (**B**) cells. **C**, **D** RT-PCR analysis was performed for 18S, RPRD1A, SOD1, SOD2, GCLC, GCLM, NQO1, HO1, ANT, PRDX4, and PRDX6 in MHCCLM3-NC and MHCCLM3-shRPRD1A (**C**), MHCCLM3-vector and MHCCLM3-RPRD1A (**D**) cells. **E** Western blotting analysis showed the levels of NRF2, GCLC, GCLM, NQO1, RPRD1A, and GAPDH in control and RPRD1A overexpressed MHCCLM3 cells. **F** RT-PCR analysis was performed to detect the expression of NRF2 in MHCCLM3-NC, MHCCLM3-shRPRD1A, MHCCLM3-vector and MHCCLM3-RPRD1A cells. **G** Western blotting analysis showed the levels of NRF2, Keap1, p62, RPRD1A, and GAPDH in MHCCLM3-NC, MHCCLM3-shRPRD1A, MHCCLM3-vector and MHCCLM3-RPRD1A cells. **H** Western blotting and quantification of NRF2 expression in lysates from MHCCLM3 cells treated with 25 μM cycloheximide for the indicated time with differentially expressed RPRD1A**. I**, **J** Ubiquitination levels of NRF2 were detected by immunoprecipitation and western blotting in MHCCLM3-NC and MHCCLM3-shRPRD1A (**I**), MHCCLM3-vector and MHCCLM3-RPRD1A (**J**) cells transfected with pcDNA3.1-HA-Ub plasmid and treated with 10 μM MG132 for 12 h. **K** Immunoprecipitation analysis to detect the binding amount of Keap1 and NRF2 under the condition of RPRD1A knockdown or overexpression. Lysates were incubated with Keap1 antibody or control immunoglobulin G and protein A&G magnetic beads overnight; western blotting was used to detect the expression of NRF2 and Keap1. **L**, **M** Ubiquitination levels of p62 were detected by immunoprecipitation and western blotting in MHCCLM3-NC and MHCCLM3-shRPRD1A (**L**), MHCCLM3-vector and MHCCLM3-RPRD1A (**M**) cells transfected with pcDNA3.1- HA-Ub plasmid and treated with 10 μM MG132 for 12 h. The *p* values were determined by a two-tailed t-test. *p < 0.05, **p < 0.01, ***p < 0.001, ns, not significant. Results are representative of at least three independent experiments.

Given that RPRD1A positively regulates the antioxidant capacity of HCC cells, we employed dual-luciferase reporter gene assays to examine whether RPRD1A influenced the ARE element activities. In HCC cells, ARE-luciferase activity was decreased under oxidative stress when RPRD1A was knocked down (Fig. 4A), while overexpression of RPRD1A significantly enhanced ARE-luciferase activity at baseline and continuously increased under H_2_O_2_ treatment (Fig. 4B). ARE regulates the transcription of a series of antioxidant enzymes, therefore we examined the expression of several antioxidant enzymes in cells in which RPRD1A was interfered or overexpressed. The mRNA levels of superoxide dismutase 1 (SOD1), SOD2, glutamate-cysteine ligase catalytic subunit (GCLC) and modifier subunit (GCLM), adenine nucleotide translocator (ANT), NAD(P)H quinone oxidoreductase l (NQO1), heme oxygenase 1 (HO1), peroxiredoxin 4 (PRDX4) and peroxiredoxin 6 (PRDX6) were positively regulated by RPRD1A (Fig. 4C, D), as well the protein level of GCLC, GCLM and NQO1 (Fig. 4E). As most of these genes are transcriptional targets of NRF2 (MacLeod et al., 2009), and NRF2 protein could be affected by RPRD1A under H_2_O_2_ treatment (Fig. 3J). We further investigate the relationship between RPRD1A and NRF2. Real-time PCR and western blotting assays revealed that RPRD1A regulated NRF2 at the protein level, but not the RNA level (Fig. 4F, G), suggesting that RPRD1A may affect NRF2 protein stability. To evaluate this hypothesis, we treated HCC cells with the protein synthesis inhibitor, cycloheximide (CHX). As expected, the NRF2 protein level gradually decreased in control cells after CHX treatment in a time-dependent manner; however, RPRD1A overexpression markedly attenuated while RPRD1A knockdown accelerated the degradation of NRF2 (Fig. 4H). We then analyzed the ubiquitination status of NRF2 in RPRD1A differentially expressed cells. The poly-ubiquitination of NRF2 increased in RPRD1A-interfered cells (Fig. 4I), while decreased in RPRD1A-overexpressed cells (Fig. 4J). As Keap1was the key regulator for NRF2 ubiquitination and degradation, we further evaluated whether RPRD1A could modulate interaction between Keap1 and NRF2. Co-immunoprecipitation (Co-IP) assay revealed that RPRD1A inhibited the association of Keap1 and NRF2 (Fig. 4K). Since the stabilization of NRF2 could be prolonged by p62-dependent sequestration of Keap1, we turned to explore the effect of RPRD1A on p62. We transfected HA-ubiquitin plasmid into RPRD1A differentially expressed cells and detected the ubiquitination of p62 by co-immunoprecipitation. As a result, RPRD1A decreased the ubiquitination of p62 (Fig. 4L, M). Altogether, these data demonstrated RPRD1A inhibited the ubiquitin-proteasome degradation of NRF2 by interrupting the p62- Keap1-NRF2 axis.

### RPRD1A competitively binds to TRIM21 to decrease the p62 ubiquitination and NRF2 degradation

**Fig. 5:**
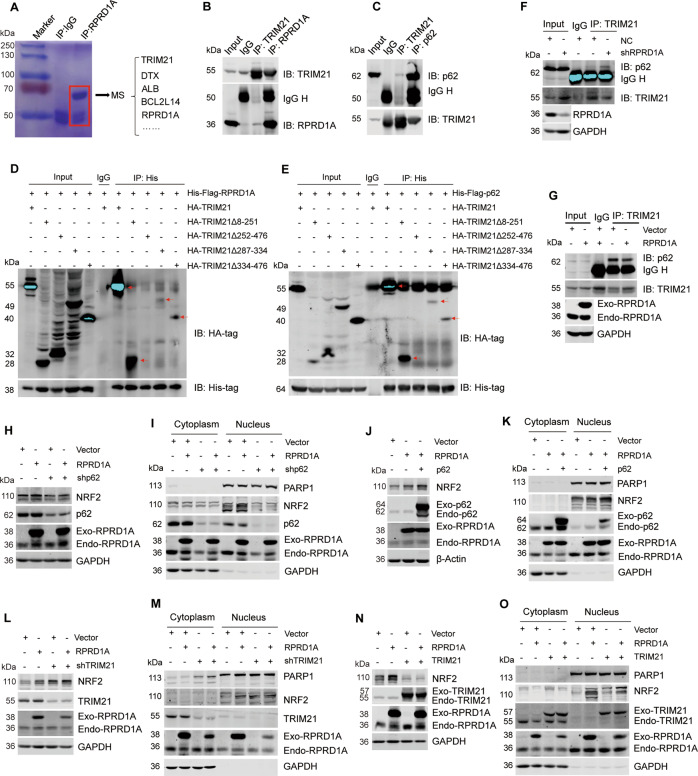
RPRD1A competitively binds to TRIM21 to decrease the p62 ubiquitination and NRF2 degradation. **A** Potential protein interactions were analyzed by mass spectrometry. Immunoprecipitation was performed in MHCCLM3 cells with antibody against RPRD1A, and then isolated by SDS-PAGE and stained with coomassie brilliant blue. All indicated protein bands were excised for mass spectrometric analysis. **B** The endogenous interaction between RPRD1A and TRIM21. MHCCLM3 cells were subjected to co-IP assays using TRIM21 or RPRD1A antibody. The abundance of RPRD1A and TRIM21 in precipitate was detected by western blotting. **C** The endogenous interaction between p62 and TRIM21. MHCCLM3 cells were subjected to co-IP assays using p62 or TRIM21 antibody. **D** His-Flag-RPRD1A and HA-tagged TRIM21 full-length or truncation mutants were expressed in HEK293T cells for 48 h. Cell lysates were subjected to co-IP assays using His-tag antibody. **E** His-Flag-p62 and HA-tagged TRIM21 full-length or truncation mutants were expressed in HEK293T cells for 48 h. Cell lysates were subjected to co-IP assays using His-tag antibody. **F**, **G** Co-IP assays to detect the interaction between TRIM21 and p62 under the condition of RPRD1A knockdown (**F**) or overexpression (**G**). **H**, **I** Western blotting to detect the protein levels of NRF2 from the whole cell lysates (**H**), cytoplasm and nuclear fractions (**I**). Empty vector or His-Flag-RPRD1A plasmid was transfected into the control and p62-knockdown MHCCLM3 cells. **J**, **K** Western blotting to detect the protein levels of NRF2 from the whole-cell lysates (**J**), cytoplasm and nuclear fractions (K). Empty vector, His-Flag-RPRD1A, and p62 plasmids were transfected into MHCCLM3 cells. **L**, **M** Western blotting to detect the protein levels of NRF2 from the whole-cell lysates (**L**), cytoplasm and nuclear fractions (**M**). Empty vector and His-Flag-RPRD1A were transfected into the control and TRIM21-knockdown MHCCLM3 cells. **N**, **O** Western blotting to detect the protein levels of NRF2 from the whole-cell lysates (**N**), cytoplasm and nuclear fractions (**O**). Empty vector, His-Flag-RPRD1A and HA-TRIM21 were transfected into the MHCCLM3 cells.

To investigate the mechanism of RPRD1A on stabilizing NRF2 and p62 protein, the immunoprecipitation by RPRD1A antibody from HCC cell lysate was detected by mass spectrometry (Fig. 5A and supplementary file 2). TRIM21, an E3 ubiquitin ligase, was identified to associate with RPRD1A (Fig. 5A). We further carried out co-IP assays and confirmed the endogenous interaction between RPRD1A and TRIM21 (Fig. 5B). A previous study has reported that TRIM21 interacts with p62 and medicates its ubiquitylation, which decreasing p62-dependent sequestration of Keap1 [13]. In our data, we verified the interaction between TRIM21 and p62 in HCC cells (Fig. 5C). More importantly, we discovered RPRD1A knockdown enhanced the TRIM21-mediated p62 ubiquitylation (Fig. S2A). An important function of p62 is to retain proteins in aggregates (sequestosomes) for subsequent autophagic degradation and Keap1 is one of the client proteins. Thus, we further detected the effects of RPRD1A on p62 aggregation and Keap1 sequestration. We discovered that RPRD1A overexpression enhanced p62 oligomerization, while RPRD1A knockdown attenuated the effect (Fig. S2B). Moreover, RPRD1A knockdown decreased the ability of p62 to sequestrate Keap1 (Fig. S2C).

Given that both RPRD1A and p62 can interact with TRIM21, we suspected that there might be a competitive interaction as RPRD1A affects the p62 ubiquitylation. We transfected a series of truncated constructs of TRIM21 into HEK293T cells together with RPRD1A or p62 plasmid to perform co-IP assays. The results showed the deletion of TRIM21 252-476 region, not the deletion of TRIM21 287–334 or 334–476 region, abolished the interaction with RPRD1A (Fig. 5D). Similar results were observed in the co-IP assay with p62, indicating that both RPRD1A and p62 interact with the 252–287 region of TRIM21 (Fig. 5E). Moreover, we found that RPRD1A knockdown increased the interaction of TRIM21 and p62 (Fig. 5F), while RPRD1A overexpression decreased their interaction (Fig. 5G). Our data supported the notion that RPRD1A and p62 might competitively interact with TRIM21. Furthermore, we verified the possible effect of RPRD1A-TRIM21-p62 interaction on regulating NRF2 abundance. Indeed, p62 knockdown attenuated the accumulation and nuclear translocation of NRF2 protein induced by RPRD1A overexpression (Figs. 5H, 5I), while p62 overexpression caused the opposite effects (Fig. 5J, K). Similarly, TRIM21 knockdown strengthened the up-regulation and nuclear translocation of NRF2 protein induced by RPRD1A (Fig. 5L, M), while TRIM21 overexpression counteracted these effects (Fig. 5N, O). Taken together, these data demonstrated that RPRD1A competitively binds to TRIM21, increasing the formation of p62 oligomerization and sequestration for Keap1, which resulted in the stabilization of NRF2 protein.

### RPRD1A induced ROS inhibition correlates with chemotherapeutic resistance

**Fig. 6:**
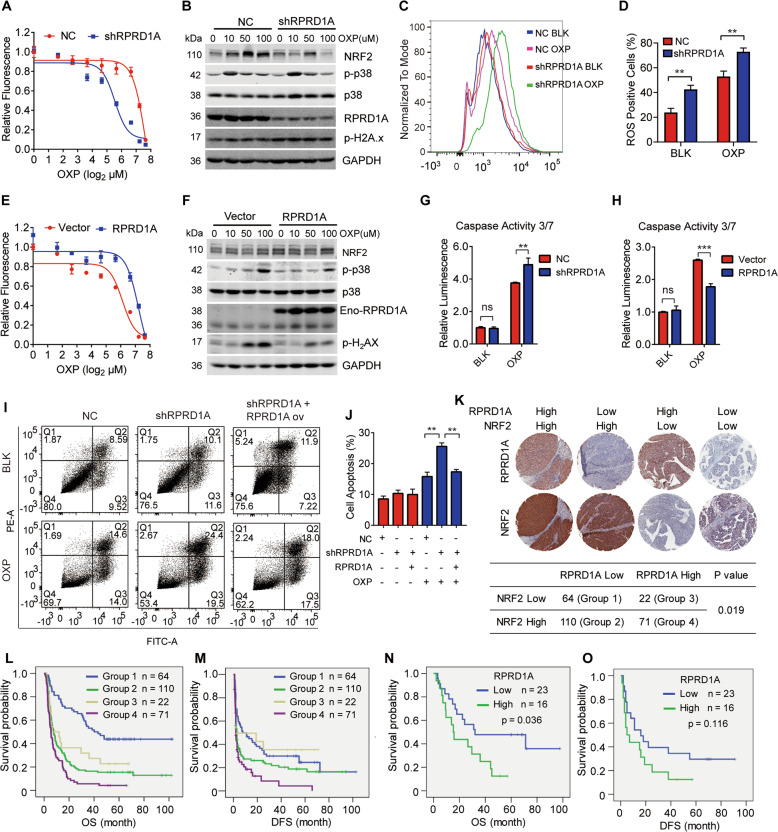
RPRD1A induced ROS inhibition correlates with chemotherapeutic resistance. **A** Cell survival of MHCCLM3 NC and shRPRD1A cells exposed to serial dilutions of OXP for 60 h. **B** Western blot of lysates generated from MHCCLM3-NC and -shRPRD1A cells treated with OXP as indicated. **C** ROS levels were detected by flow cytometry in MHCCLM3 NC and shRPRD1A cells treated with 50 μM OXP or control for 4 h. **D** Quantification of the ROS levels in (**C**). **E** Cell survival of control and MHCCLM3-RPRD1A cells exposed to serial dilutions of oxaliplatin for 60 h. **F** Western blotting of lysates generated from control and MHCCLM3-RPRD1A cells treated with OXP as indicated. **G**, **H** Caspase 3/7 assay was performed to detect the activity apoptosis of MHCCLM3-NC and MHCCLM3-shRPRD1A (**G**), control and MHCCLM3-RPRD1A (**H**) treated with 50 μM OXP for 24 h. **I** Annexin V and PI staining analysis were performed to test cell apoptosis of MHCCLM3-NC, MHCCLM3-shRPRD1A and RPRD1A supplemental cells treated with or without 100 μM OXP for 6 h. **J** Histogram was the quantification. **K** Representative images of RPRD1A and NRF2 staining in HCC specimens. Group 1, low NRF2 and low RPRD1A expression (*n* = 64); group 2, high NRF2 and low RPRD1A expression (*n* = 110); group 3, low NRF2 and high RPRD1A expression (*n* = 22); and group 4, high NRF2 and high RPRD1A expression (*n* = 71). The cross table showed the distribution of RPRD1A and NRF2 expression in HCC samples (*n* = 267); the Pearson λ^2^ correlation coefficient and *p* value are shown. **L**, **M** Kaplan–Meier analysis of OS (**L**) and DFS (**M**) in HCC patients according to RPRD1A and NRF2 expression. **N**, **O** Kaplan–Meier analysis of OS (**N**) and DFS (**O**) in HCC patients with TACE according to RPRD1A expression. In panels (**D**), (**H**), (**J**), the *p* values were determined by a two-tailed t-test. In panels **L**–**O**, the *p* values were determined by the log-rank test. *p < 0.05, **p < 0.01, ***p < 0.001. Results are representative of at least three independent experiments.

Platinum drugs treatment induces ROS and DNA damage, and leads to cell death [16]. We then examined the effect of RPRD1A on platinum-induced cell death. Cell survival assays showed that RPRD1A knockdown facilitated the chemosensitivity to oxaliplatin (OXP) (Fig. 6A). Western blotting analysis revealed that RPRD1A depletion increased p-H2AX and p-p38 levels under oxaliplatin treatment (Fig. 6B), indicating severe oxidative and genotoxic stress in cells. Indeed, RPRD1A knockdown increased ROS levels induced by oxaliplatin (Fig. 6C, D). On the contrary, RPRD1A overexpression caused the opposite effect, promoting cell survival and attenuating DNA damage (Fig. 6E, F). This result was further confirmed by the detection of activated caspase-3/7 that RPRD1A attenuated the caspase activities under oxaliplatin treatment (Fig. 6G, H). Moreover, RPRD1A knockdown promoted cell apoptosis while re-expressing RPRD1A rescued this phenomenon (Fig. 6I, J). Together, these data indicated that RPRD1A helped cancer cells resistance to OXP-induced oxidative stress and cell death.

**Fig. 7:**
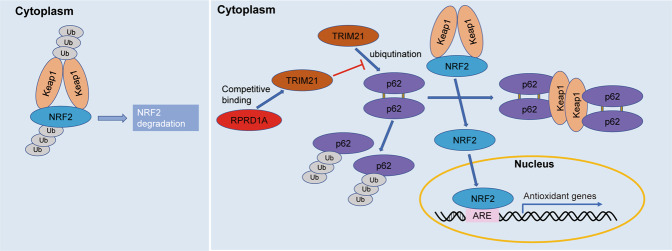
A proposed model illustrating the mechanism of RPRD1A stabilizes NRF2. Under normal conditions, Keap1 interacts with NRF2 and ubiquitinates it to degrade via the ubiquitin-proteasome pathway. In response to oxidative stress, RPRD1A competes with p62 to bind TRIM21 and inhibits ubiquitination of p62 by TRIM21. The process releases p62 and causes its self-oligomerization, leading to the segregation and degradation of Keap1, which frees and stabilizes NRF2. Then, NRF2 translocates to the nucleus where it binds the antioxidant response element (ARE) and activates the transcription of antioxidant genes.

Next, we explored the correlation among RPRD1A, NRF2, and OXP chemosensitivity in the HCC cohort. Based on RPRD1A and NRF2 expression in tissue microarrays, patients in this cohort were classified into the indicated four groups (Fig. 6K, top). Statistical analysis revealed a positive correlation between RPRD1A and NRF2 expression (Fig. 6K, bottom). Kaplan–Meier survival analysis showed that patients in group 1 (low RPRD1A expression and low NRF2 expression) had the longest OS and DFS, while group 4 (high RPRD1A expression and high NRF2 expression) had the shortest OS and DFS among all groups (Fig. 6L, M), indicating that the combination of RPRD1A and NRF2 had practical prognosis values. In clinical practice, OXP was widely used in transarterial chemoembolization (TACE), which is one of the most common adjuvant managements for HCC. TACE prevented recurrence and prolonged the survival of HCC patients postoperatively [17]. Consistent with the above data, we observed that patients with low RPRD1A expression in tumors had a better response to adjuvant TACE treatment (Fig. 6N, O). Altogether, our data indicated that RPRD1A stabilizes NRF2 by competitively binding with TRIM21 for p62, which further leads to the resistance to ROS and platinum drugs (Fig. 7).

## Discussion

In oxidative stress conditions, p62 recruits Keap1 from the cytoplasmic Keap1-NRF2 complex [18]. As a result, the free NRF2 translocates into the nucleus as a transcription factor, inducing the expression of a battery of NRF2 target genes encoding antioxidant enzymes [10]. We demonstrated here RPRD1A was highly expressed in HCC primary tumors and PVTT tissues. Elevated RPRD1A competitively interrupted the association between TRIM21 and p62 and subsequently inhibited the ubiquitination of p62, leading to more Keap1 sequestration and subsequently stabilization of NRF2. This regulation helps combating excessive intracellular ROS, which reduces oxidative stress-induced cell death and further contributes to the progression of HCC.

RPRD1A, also called P15RS, regulates G1/S cell cycle progression and suppresses Wnt and β-catenin signaling via interactions with the class I lysine deacetylase HDAC2 and transcription factor 4 (TCF4) [19]. RPRD1A was demonstrated as a tumor suppressor [20]. To our knowledge, there have been no reports on the role of RPRD1A in liver cancer progression. In our study, we identified RPRD1A maintained p62 sequestration function towards Keap1 through interacting with TRIM21 in HCC cells. TRIM21 is a RING finger domain-containing E3 ligase that belongs to the family of the tripartite motif (TRIM) family [21]. TRIM21 directly interacts with and ubiquitylates p62 at residue K7, which inhibits p62 dimerization and sequestration function [13]. There seems to be some contradiction between our research and previous reports about the function of RPRD1A in the tumor, indicating the different tumor types may possess different function of specific gene. Furthermore, previous investigations pay attention to the function of RPRD1A in the nucleus, while our study mainly focused on its role in the cell cytoplasm.

Drug resistance remains a major obstacle to the treatment of cancer. It has previously been suggested that cellular ROS is critical for chemotherapeutic response. Platinum-based drugs strongly generate reactive chemicals, including ROS, that either directly triggers apoptosis or exacerbate drug-induced DNA damage [22]. Antioxidants have been demonstrated to confer cancer cells with not only promoted anchorage-independent growth but also increased resistance to chemotherapy. Here, we validated that the effect of RPRD1A on drug resistance can also be achieved by an anti-ROS function. Meanwhile, we found that the anti-ROS activity of RPRD1A is largely dependent on its activity by binding to TRIM21, leading to more capture of Keap1 by more p62. Moreover, knockdown of RPRD1A endowed HCC cells with elevated sensitivity to conventional platinum-based drugs.

## Conclusions

In summary, our data reveal that RPRD1A is highly expressed in HCC tumors and correlated with aggressive clinicopathological features. RPRD1A competes with p62 for binding to TRIM21, thereby stabilizing p62 and enhancing the aggregation between p62 and Keap1, further promoting the accumulation and nuclear translocation of NRF2 to cope with oxidative stress. Therefore, the RPRD1A-TRIM21-p62 axis may be a possible therapeutic target to improve the sensitivity of HCC cells to chemotherapeutic drugs.

## Materials and methods

### Human tumor samples

Hepatocellular carcinoma, para-carcinoma specimens, and patients’ clinical data were obtained from the Eastern Hepatobiliary Surgery Hospital, Second Military Medical University, Shanghai, China, between January 2010 and December 2016, with the approval of the Eastern Hepatobiliary Surgery Hospital Research Ethics Committee. Informed consent was obtained from all patients. All patients’ diagnosis was confirmed by pathological analysis.

### Cell lines and cell culture

The human hepatocellular carcinoma cell lines MHCCLM3 and Huh7 were obtained from the Shanghai Cell Resource Center of the Chinese Academy of Sciences. Human embryonic kidney cell line HEK293T was purchased from ATCC. All cells were maintained in Dulbecco’s modified Eagle medium (DMEM) supplemented with 10% fetal Bovine Serum (Biological Industries, Israel), and maintained at 37°C in an atmosphere of humidified air containing 5% CO_2_.

### Plasmids, DNA transfection and lentivirus

Vector, pcDNA3.1-HA-Ub, pEnter-His-Flag-SQSTM1/p62, pEnter-His-Flag-RPRD1A, ptt5-HA-TRIM21, ptt5-HA-TRIM21Δ8-251, ptt5-HA-TRIM21Δ252-476, ptt5-HA-TRIM21Δ287-334, ptt5-HA-TRIM21Δ334-476 plasmids were purchased from Vigene Bioscience. Cells were transfected into plasmids with jetPEI DNA Transfection Reagent (Polyplus) according to the manufacturer’s instructions. The lentiviruses that knocked down RPRD1A and control NC were purchased from Genechem. The shRNA sequences of RPRD1A were as follows: shRPRD1A1#, 5′- GGCGGCAGAAAUAGAUGAUTT-3′, shRPRD1A2#, 5′-GCAUGUUUCAAGUGAAACUTT-3′. The shRNA sequences of TRIM21 were: 5′-GCAGGAGUUGGCUGAGAAG-3′. The shRNA sequences of p62 were: 5′-GCAUUGAAGUUGAUAUCGAU-3′. The negative control sequences were: 5′-UUCUCCGAACGUGUCACGUTT-3′. HCC cells were infected with lentivirus for 48 h and the knockout effect was detected by western blot.

### RNA extraction, cDNA synthesis and Real-time quantitative PCR

Total RNA from cultured cells and tissue specimens was extracted using TRIZOL reagent (Invitrogen, USA). A total of 2 μg per sample RNA was used for complementary DNA synthesis using Oligo (dT)15 primers reagents kit (Promega, USA). Real-time PCR (RT-PCR) was carried out in LightCycler^@^ using an SYBR^@^ Green Master kit (Roche, Switzerland). The primer sequences are as follows: 18S, 5′-CGGCTACGACATCCAAGGAA-3′ and 5′-GCTGGAATTAGCGCGGCT-3′; hRPRD1A, 5′-ATGGTAGAGGATGCGTGTATGT-3′ and 5′-AAGGGCTTCCTTTTGACAACG-3′; hNRF2, 5′-CACATCCAGTCAGAAACCAGTGG-3′ and 5′-GGAATGTCTGCGCCAAAAGCTG-3′; hSOD1, 5′-AGGGCATCATCAATTTCGAGC-3′ and 5′-GCCCACCGTGTTTTCTGGA-3′; hSOD2, 5′-AACCTCAGCCCTAACGGTG-3′ and 5′-AGCAGCAATTTGTAAGTGTCCC-3′; hANT, 5′-TCCCCACCCAAGCTCTCAA-3′ and 5′-GTCCAGCGGGTAGACAAAGC-3′; hGCLC, 5′-AGAGAAGGGGGAAAGGACAAAC-3′ and 5′-AAGTTATTGTGCAAAGAGCCTGAT-3′; hGCLM, 5′-TCAGGGAGTTTCCAGATGTCTTG-3′ and 5′-TGAAGCAATGATCACAGAATCCA-3′; hNQO1, 5′-GCAGTTTCTAAGAGCAGAAC-3′ and 5′-GTAGATTAGTCCTCACTCAGCCG-3′; hPRDX4, 5′-GCAAAGCGAAGATTTCCAAG-3′ and 5′-GGCCAAATGGGTAAACTGTG-3′; hPRDX6, 5′-GCATCCGTTTCCACGACT-3’ and 5′-TGCACACTGGGGTAAAGTCC-3′; HO1, 5′-GGGCTAGCATGCGAAGTGAG-3′ and 5′- AGACTCCGCCCTAAGGGTTC -3′.

### Western blotting, co-immunoprecipitation and antibodies

Cells and tissues were lysed in RIPA buffer for 20 min on ice and centrifuged at 12,000 rpm 4 °C for 15 min. Protein concentrations were measured using the bicinchoninic acid assay (Thermo Fisher Scientific, USA). 20–100 μg proteins were separated by SDS-polyacrylamide gel and transferred to nitrocellulose membranes. Western blotting was performed using specific primary antibodies overnight at 4°C. After washing with TBST buffer (10 mM Tris-HCL, PH7.4, 100 mM NaCl, 1‰ Tween 20), immune complexes were incubated with the fluorescein-conjugated secondary antibody and then detected using an Odyssey fluorescence scanner (Li-Cor, Lincoln, Neb).

For co-immunoprecipitation experiments, cell lysates were measured and incubated with 2 μg anti-RPRD1A (Proteintech Group, China, Cat# 23652-1-AP), anti-NRF2 (Proteintech Group, China, Cat# 16396-1-AP), anti-Keap1 (Proteintech Group, China, Cat# 10503-2-AP), anti-p62 (Proteintech Group, China, Cat# 18420-1-AP), anti-TRIM21 (Proteintech Group, China, Cat# 67136-1-Ig), anti-Flag (Proteintech Group, China, Cat# 80010-1-RR), or normal mouse (Santa Cruz Biotechnology, USA, Cat# sc-2025) or rabbit immunoglobulin G (Santa Cruz Biotechnology, Cat# sc-66931) for 8 h at 4 °C, followed by the addition of protein A/G Plus-Agarose (Santa Cruz Biotechnology, Cat# sc-2003) for another 4 h. Proteins were eluted into protein loading buffer, followed by western blotting.

Anti-HA (Cat# 51064-2-AP), Anti-GCLC (Cat# 12601-1-AP), anti-GCLM (Cat# 14241-1-AP), anti-GAPDH (Cat# 10494-1-AP), and anti-NQO1 (Cat# 11451-1-AP) were purchased from Proteintech Group, Inc. Anti-p-H_2_AX (Cat# AP0687) was purchased from Abclonal Inc, China. Anti-p-p38 (Cat# 4511) and anti-p38 (Cat# 8690) were purchased from Cell Signaling Technology, USA.

### Histology and immunohistochemistry

Formalin-fixed paraffin-embedded tissues were sectioned (5 mm) and stained with hematoxylin and eosin (H&E) for histological analysis or used for immunohistochemistry (IHC). For IHC, primary antibodies were: anti-NRF2 (1:200), anti-RPRD1A (1:150), anti-GPC3 (ABclonal, Cat# A13988, 1:200) and anti- Heparanase 1 (ABclonal, Cat# A16488, 1:200). In all cases, heat-mediated antigen retrieval was performed. The binding of antibody was detected using diaminobenzidine substrate (Dako, Denmark).

### Cell proliferation

Cell proliferation was measured using CCK8 reagent (Roche) according to the manufacturer’s instructions. Cells were seeded into 96-well plates at a density of 3000 cells/well. When cells were cultured for the indicated time, CCK8 reagent was added. All experiments were performed in triplicate.

### Plate clone formation assay

For plate clone formation assay experiment, MHCCLM3-NC and MHCCLM3-shRPRD1A, Huh7-NC and Huh7-shRPRD1A cells were resuspended in DMEM supplemented with 10% FBS and seeded to 6-well plates (Corning) for 5000 cells/well for two weeks. Then the cells were fixed with 4% formaldehyde and stained with 0.1% crystal violet for imaging.

### Transwell assay

Cell migration assays were measured with transwell cell culture chambers. MHCCLM3-NC and MHCCLM3-shRPRD1A, Huh7-NC and Huh7-shRPRD1A cells were resuspended in serum-free DMEM to a final concentration of 8 × 10^4^ cells/mL and were seeded to the upper compartment of transwell cell culture chambers (Corning Inc, 6.5 mm diameter, 8.0 μM pore size). The lower chamber was filled with DMEM supplemented with 10% FBS. After culture for the indicated time, the transwell cell culture chambers were fixed with 4% formaldehyde and scraped off by a cotton swab to move the cells inside the chambers and then stained with 0.1% crystal violet, followed by microscopic imaging.

### Immunofluorescence assay

The cells were seeded to the confocal dish, fixed for the indicated time, perforated with 0.2% Triton X-100 and sealed with 1% BSA, then incubated with primary antibodies overnight and washed with PBS for three times, then incubated with secondary antibodies and DAPI, and finally imaged using a Leica SP8 confocal microscope equipped with a ×63 oil immersion objective.

### Subcutaneous tumor-burdened and Pulmonary metastases

Female nude mice aged 6 weeks were randomly divided into two groups, approximately 2 × 10^6^ cells in 0.1 mL PBS were injected subcutaneously into the right flank of the female nude mice aged 6 weeks. Once the subcutaneous tumor reached 1–1.5 cm in diameter, the tumor was harvested. Tumor volume was calculated as follows: *V* (mm^3^) = width^2^ (mm^2^) × length (mm)/2. All experiments were performed with at least four mice in each group and repeated three times. For in vivo metastasis assay, female nude mice aged 6 weeks were randomly divided into two groups and injected with 1 × 10^6^ cells in 0.1 mL PBS through the caudal vein. Approximately three months later, mice were sacrificed and lung metastases were observed. No samples or animals were excluded from the analyses.

### Assessment of cell death and ROS level

Cells were treated as designed, followed by incubation with PI (Sigma-Aldrich, Shanghai, CA) and ROS Assay Kit (Beyotime Biotechnology, Shanghai, CA) for 20 min respectively, and then were analyzed the cell death and the level of ROS by fluorescence microscope and the flow cytometry (BD, New Jersey, USA).

### Total antioxidant capacity assay

Cells were cultured and stimulated with PBS or 0.5 mM H_2_O_2_ for 4 h. Total Antioxidant Capacity was measured with the T-AOC Assay kit (Total Antioxidant Capacity Assay kit with a Rapid ABTS method) purchased from Beyotime according to the manufacturer’s instructions.

### Luciferase assay

Luciferase assays were performed in 96-well plates using MHCCLM3 and Huh7 cells transfected with ARE-luc reporter plasmid along with an internal control pRL-TK, after transfection of 36 h, luciferase activity was determined by the Dual-luciferase Assay System (Promega). Firefly luciferase activity was normalized against Renilla luciferase activity.

### Nuclear and cytoplasmic proteins extraction assay

Nuclear and Cytoplasmic proteins extraction assays were performed using NE-PER Nuclear and Cytoplasmic Extraction Reagents kit (Thermo Fisher, Shanghai, China) according to the manufacturer’s instructions. Briefly, (1) harvest the adherent cells and transfer 1 × 10^6^ cells to a 1.5 mL microcentrifuge tube. (2) Add ice-cold CER I and CER II, respectively. Vortex the tube vigorously. (3) Centrifuge and collect the cytoplasmic extract. (4) Add ice-cold NER to the insoluble fraction, vortex the sample for 15 s every 10 min, for a total of 40 min. (5) Centrifuge and collect the nuclear extract. The cytoplasmic and nuclear extracts were further detected by immunoblot.

### Statistical analysis

No statistical method was used to predetermine sample size. GraphPad Prism software 8.0 was used to conduct the statistical analysis of all data. Data were presented as mean ± SD. An unpaired two-tailed Student’s *t* test was used to define statistical significance when two groups were compared. For all cell survival and tumor growth experiments, unless otherwise indicated, more than three technical replicates were performed and data were assessed by two-way analysis of variance (ANOVA). IC50 values were calculated by nonlinear regression analysis of dose-response data in Prism software. Kaplan–Meier survival curves were generated using SPSS Statistics 20 software, and the log-rank test was performed to assess the statistical significance of differences between the two groups. *p* < 0.05 was considered statistically significant.

## Supplementary information

Figure S1:RPRD1A promoted cell proliferation in Huh7 cells

Figure S2:RPRD1A promoted p62 aggregation and the sequestration for Keap1.

supplementary file 1:high-throughput sequence

supplementary file 2:mass spectrometric data

Extended Data 1: AJ-checklist

## Data Availability

All data needed to evaluate the conclusions in the paper are presented in the paper and/or in the supplementary data files.
