# Supplementary figures and images for: RPRD1A stabilizes NRF2 and aggravates HCC progression through competing with p62 for TRIM21 binding
Source: Cell Death Dis. 2021 Dec 17;13(1):6. doi: 10.1038/s41419-021-04447-4 (PMC8683478; doi:10.1038/s41419-021-04447-4)

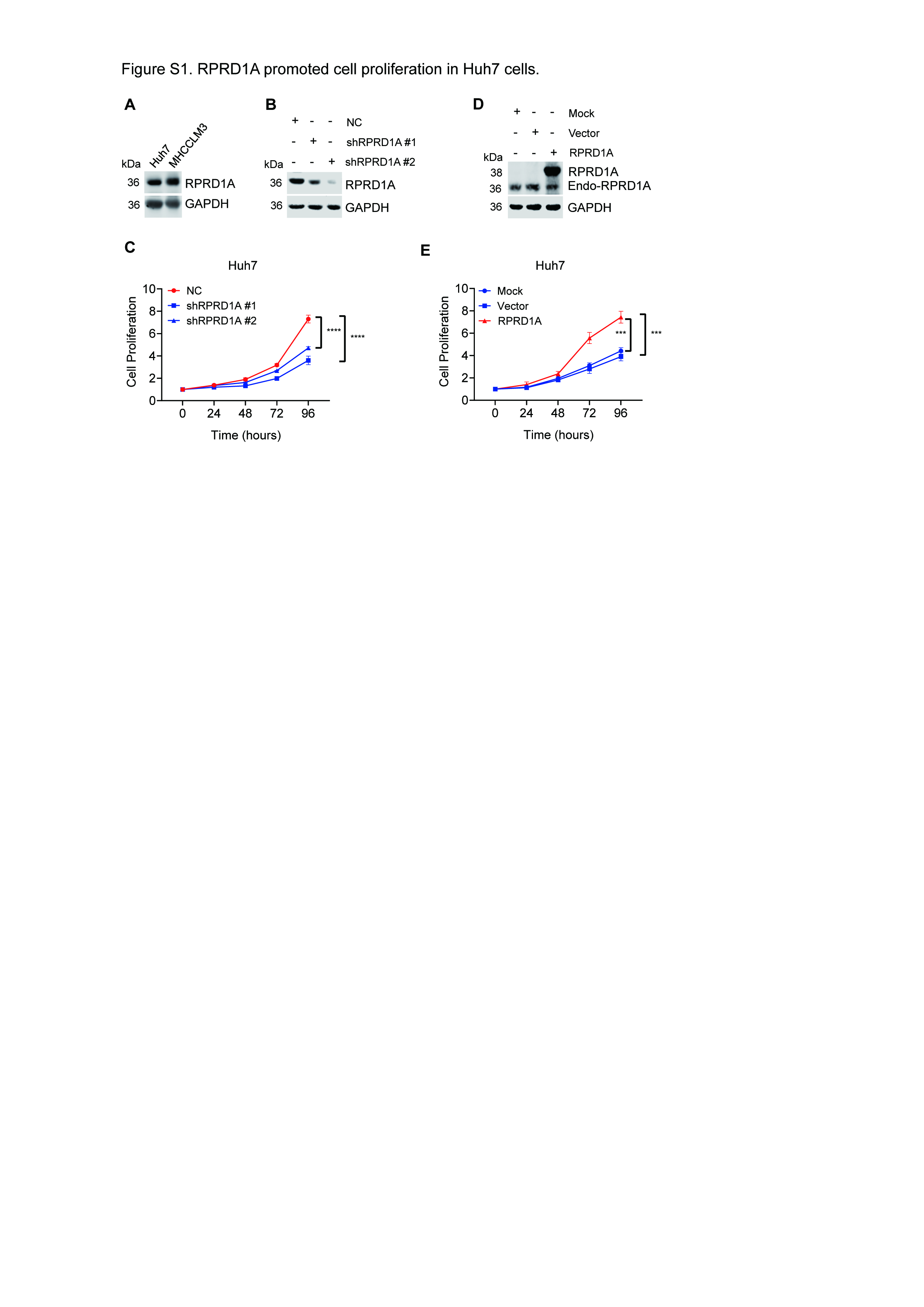

Supplement: Supplementary file 1 — Figure S1:RPRD1A promoted cell proliferation in Huh7 cells [file 41419_2021_4447_MOESM1_ESM.tif]

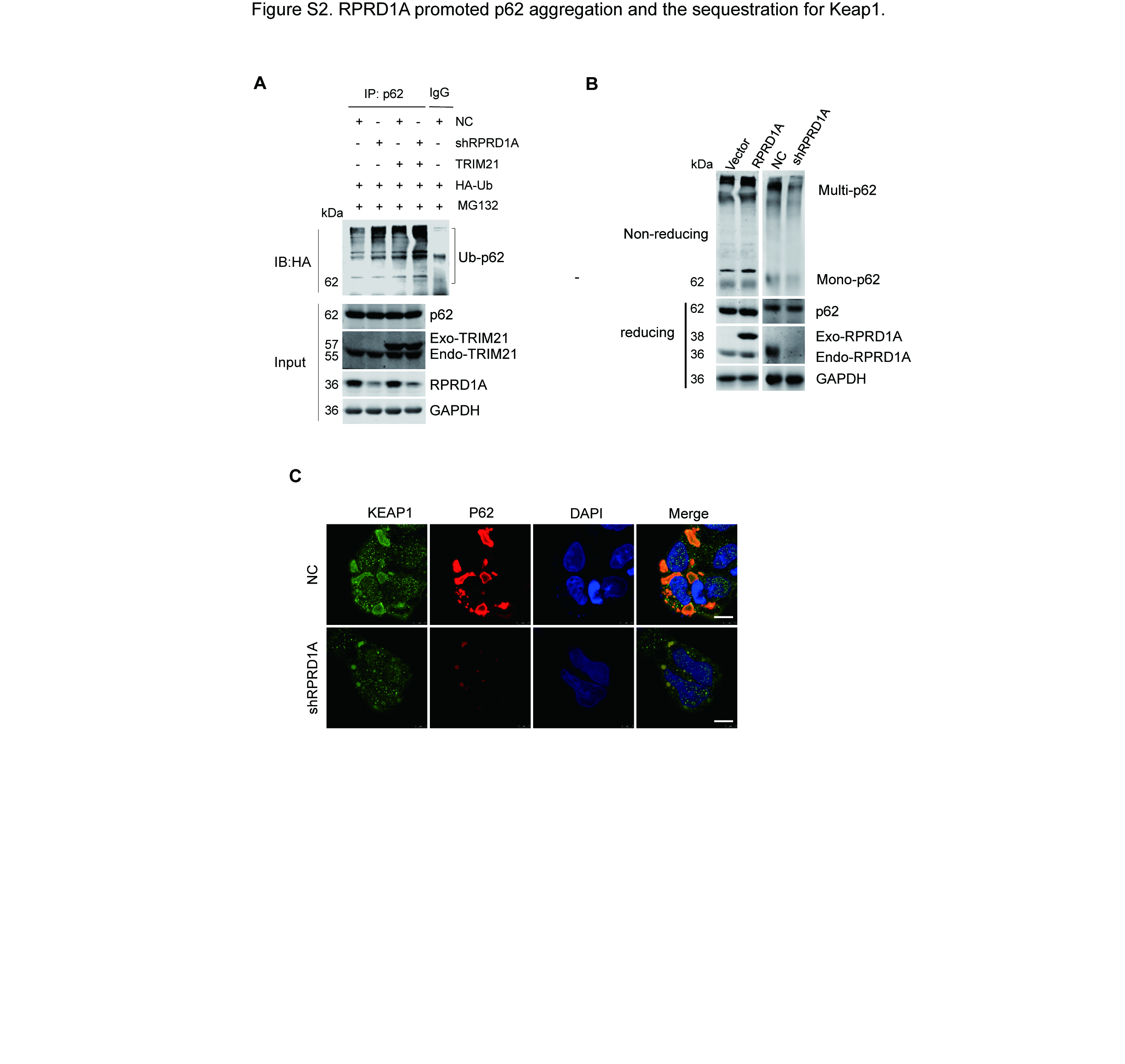

Supplement: Supplementary file 2 — Figure S2:RPRD1A promoted p62 aggregation and the sequestration for Keap1. [file 41419_2021_4447_MOESM2_ESM.tif]
